# The ENJOY MAP for HEALTH: Exercise interveNtion outdoor proJect in the cOmmunitY for older people—More Active People for HEALTHier communities: a study protocol

**DOI:** 10.1186/s12889-022-13335-1

**Published:** 2022-05-21

**Authors:** Pazit Levinger, Jeremy Dunn, Emma Abfalter, Briony Dow, Frances Batchelor, Stephanie Garratt, Neil T Diamond, Keith D. Hill

**Affiliations:** 1 National Ageing Research Institute, Royal Melbourne Hospital, PO Box 2127, Melbourne, VIC 3050 Australia; 2 Institute for Health and Sport, Victoria University, Melbourne, Australia; 3 Rehabilitation, Ageing and Independent Living Research Centre, Monash University, Melbourne, Australia; 4 Faculty of Medicine, Dentistry and Health Sciences, University of Melbourne, Melbourne, Australia; 5 School of Nursing and Midwifery, Deakin University, Waurn Ponds, Geelong, Australia; 6 Department of Physiotherapy, The University of Melbourne, Melbourne, Australia; 7 ESQUANT Statistical Consulting, Melbourne, Australia

**Keywords:** Seniors Exercise Park, Physical activity, Exercise, Built environment, Older people

## Abstract

**Background:**

Physical activity is important to maintain health in older age, with physical activity in the outdoors providing mental and physical health benefits for all age groups. One way by which older people can engage in physical activity in the outdoors is through using suitable age-friendly outdoor exercise equipment, the Seniors Exercise Park. The ENJOY MAP for HEALTH aims to evaluate the effect of the Seniors Exercise Park installation and associated capacity building activities on park visitation, park-based physical activity by older people and delivery of community physical activity programs.

**Method:**

This study is a quasi-experimental (natural experiment) with pre and post study design evaluating the effect of age-friendly outdoor spaces with specialised outdoor exercise equipment on older people’s physical activity and wellbeing in six Victorian municipalities (local governments/councils). Each council will undergo four stages (site construction and development, promotion and marketing, capacity building and training, evaluation and sustainability). Several activities and methods will be employed from stage one through stage four to evaluate the potential impact of the age-friendly outdoor spaces on physical activity and wellbeing and will comprise the following elements: site observation and equipment utilisation, face to face intercept surveys, development of an online access monitor and community building activities.

**Discussion:**

The project is expected to result in a significant change in the physical outdoor environment for the participating councils and communities whereby older people and other community members will be able to engage in safe physical and social activity programs, socialise more and hence improve the overall wellbeing of older people.

**Trial registration:**

This trial is retrospectively registered with the Australian New Zealand Clinical Trials Registry. Trial registration number ACTRN12621000965808. Date registered 23/07/2021.

## Background

The pace of population ageing in Australia is increasing rapidly. In 2017, approximately 3.8 million people (15% of Australia’s total population) were aged 65 and over, by 2057, it is projected there will be 8.8 million older people in Australia (22% of the population) [1]. Older age is often characterized by the emergence of complex health issues affecting health and wellbeing. Low levels of physical activity contributes significantly to poor physical and mental health of older Australians [2]. Maintaining adequate levels of physical activity can reduce the risk of health problems in older adults [3]. However, older people have low participation rates in physical activity programs, with only 25% of Australians aged ≥ 65 meeting physical activity guidelines [4].

Exercising outdoors has various health benefits including mental and physical health for all age groups [5]. One way by which people can engage in physical activity in the outdoors is through using outdoor exercise equipment. Outdoor exercise equipment has become quite popular in recent years with many local governments (councils) installing it within local parks or recreational spaces within their municipalities [6–8]. The availability of outdoor exercise equipment offers an important environmental infrastructure to provide opportunities for physical activity and social connectedness in public settings at no cost for residents [6, 8]. Despite the emerging popularity of outdoor exercise equipment, limited suitable outdoor exercise equipment and space are specifically designed to suit older people [9]. Older people often experience complex health problems that limit mobility and functional movement which require careful consideration for the design and inclusion of suitable outdoor exercise equipment in recreational and leisure spaces.

We have been undertaking research for the past several years involving a specialised outdoor multimodal exercise equipment, the Seniors Exercise Park, and developed and tested an outdoor physical and social activity program for older people in the community. The Seniors Exercise Park integrates multimodal exercise stations that target balance (unstable/uneven surfaces), strength, flexibility and functional movements, designed for use by older people. Building on a Randomised Controlled Trial [10, 11], our work has expanded into public community settings involving the installation of the specialised equipment in multiple communities (The ENJOY project). Participation in the ENJOY program resulted in various health benefits including improved physical function and wellbeing as well as sustained engagement in physical activity, perceived enjoyment and enhanced socialisation for older people [12, 13].

The outcomes achieved so far from the ENJOY project suggest that the Seniors Exercise Park is effective in improving older people’s physical function and wellbeing and can be an important public health infrastructure investment in promoting physical activity for older people. It was also evident that working with local governments and community in partnership can play a major role in promoting community health through physical activity [14]. Wider implementation of this initiative to include more specialised equipment in the community and hence further local governments’ engagement can potentially have greater public health benefits. In addition, effective communication, strategic planning and community and older people engagement approaches are important for successful implementation [14]. The ENJOY MAP for HEALTH builds on our previous work and the lessons learnt to better improve the built environment to promote physical activity for older people. The ENJOY MAP for HEALTH aims to evaluate the impact of the Seniors Exercise Park installation and associated capacity building activities on older people’s park visitation, park-based physical activity and delivery of community physical activity programs. The ENJOY MAP for HEALTH will involve the installation of the specialised Seniors Exercise Park equipment supported by promotion and community capacity building activities in six municipalities in Victoria, Australia.

## Methods and design

All procedures involved in this trial will be conducted in compliance with the National Statement on Ethical Human Research and the Australian Code for the Responsible Conduct of Research. Ethical approval has been obtained from Monash University Human Research Ethics Committee, Melbourne Australia (Project ID: 25499). The study was designed according to the strengthening the reporting of observational studies in epidemiology (STROBE) statement [15].

**Fig. 1:**
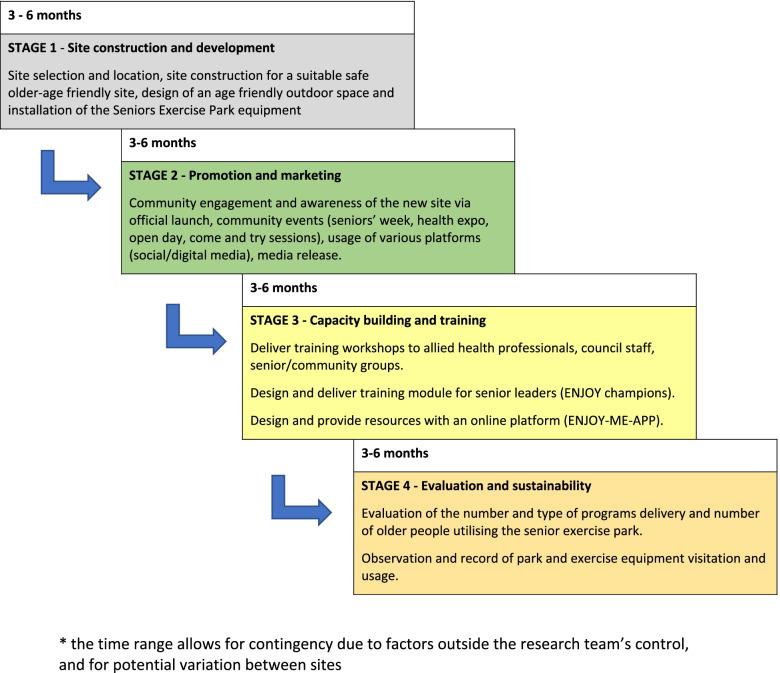
Project’s stages for each site

The ENJOY MAP for HEALTH is a quasi-experimental (natural experiment) project involving the installation of specialised seniors exercise equipment supported by promotion and community capacity building activities in six municipalities in Victoria. The project comprises four stages in each of the six participating sites and is expected to be implemented in three blocks, with staggered commencement of two sites per block. Each block will involve the creation and activation of two age friendly sites (two municipalities). Each council will undergo four stages: site construction and development, promotion and marketing, capacity building and training, evaluation and sustainability, as shown in Fig. 1.

**Fig. 2:**
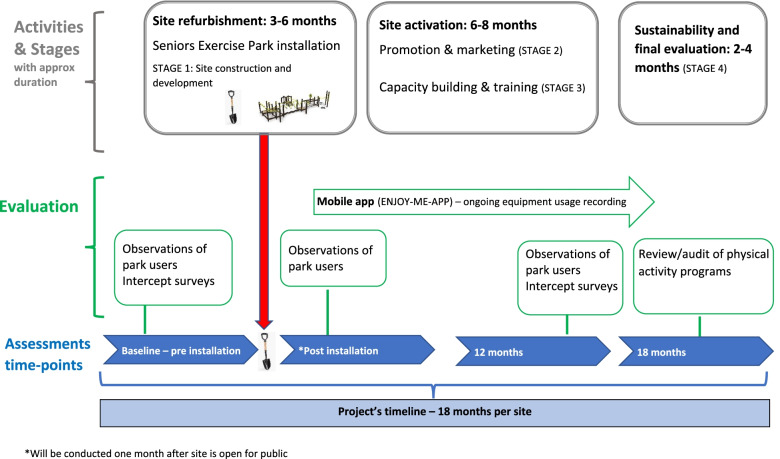
Study’s stages, activities, evaluation and timeline for each site

To address the study aims, multiple methodologies will be employed throughout the project at each site (estimated 18 months process within each site) to evaluate the impact of site refurbishment and equipment installation on park visitation and physical activity engagement and participation of older people. These will include direct observations of park users, onsite intercept surveys with park users, online access monitor platform (using an online app, *ENJOY-ME-APP’*), and review of the physical activity program offered by the participating partners and or the respective local health/leisure providers. Please refer to Fig. 2 for the project’s timeline and activities.

### Study setting and partners

In this project six councils will be involved, five in metropolitan Melbourne and one in regional Victoria as follows: Knox City Council (metropolitan), Banyule City Council (metropolitan), City of Ballarat (regional Victoria), Frankston City Council (metropolitan), City of Boroondara (metropolitan), and Bayside City Council (metropolitan).

The design of the project aims for implementation in staggered stages (in three blocks, two councils per block) where each council has nominated to undergo site refurbishment in a specific timeline based on council’s site upgrade plan. Each participating council will install the Seniors Exercise Park as part of a park/site upgrade (refurbishment) in the following block allocation:

Block one: Banyule City council (Ivanhoe Park, Ivanhoe) and City of Ballarat (Mt Pleasant Reserve, Mt Pleasant).

Block two – Bayside City Council (Thomas St Reserve, Hampton) and Knox City Council (Carrington Park, Knoxfield).

Block three – City of Boroondara (Victoria Park, Kew) and Frankston City Council (Wingham Park, Frankston).

Suitable locations have been chosen with some sites planned to undergo upgrade of play space for children in addition to the installation of the Seniors Exercise Park and other amenities (e.g., water fountain, shaded area, benches). As such there will be variation between sites around the overall site size, surrounding areas and amenities, and the additional play equipment.

#### Study population

##### Recruitment

Active promotion about the park and the overall project will be communicated through the local councils’ various marketing platforms (social media, council’s website and Facebook, flyers, local newsletter). Potential participants for the face to face intercept interview will be recruited at the site. Information about the study will be provided to residents/visitors via verbal communication as well as via hard copies of the information sheet.

##### Inclusion criteria

For the observation data collection, all park visitors will be included in the data recording. For the face-to-face intercept survey, the following inclusion criteria will be applied: (1) older adults age 60 and over, (2) adults who are able to understand basic English and have conversational English.

##### Exclusion criteria

The following exclusion criteria will be applied: (1) participants who identify themselves as less than 60 years of age and (2) who are unable to understand basic conversational English.

##### Consent

Verbal consent will be required in order to participate in the face-to-face intercept survey. The research staff will explain the study and will seek verbal agreement/consent by the participant prior to commencing the survey. A hard copy of the information sheet will be available and will be offered to potential participants should they wish to read and or maintain a copy.

### Assessments

#### Outcome measures

##### Park observation and Seniors Exercise Park equipment usage

Observation of park and exercise equipment visitation and usage will take place prior to site construction (baseline) and at two time points: one month after the site is open for public and 12 months after baseline (Fig. 2). The inclusion of two measures post-refurbishment/construction allows time for residents to become aware of the new facilities [16]. The 12 months evaluation will take place at the same time of the year as the baseline assessments to account for potential seasonal effects.

Evaluation of usage and visitations will include periodic observation (using the System for Observing Play and Recreation in Communities (SOPARC)). The SOPARC is a reliable and feasible instrument for assessing physical activity and associated contextual data in community settings [17]. It is based on momentary time sampling techniques in which systematic and periodic scans of individuals and contextual factors within pre-determined target areas in parks are made. SOPARC was designed to obtain direct information on community park use, including relevant concurrent characteristics of parks and their users. It provides an assessment of park users’ physical activity levels, gender, activity modes/types, and estimated age and ethnicity groupings. Additionally, it provides information on individual park activity areas, such as their levels of accessibility, usability, supervision, and organization. As this study focuses on the usage of the Seniors Exercise Park, we will also collect additional data (not included in the original SOPARC protocol) by coding the interaction with the outdoor exercise equipment (i.e., ‘using equipment as intended’ or ‘playing/looking/sitting’ on the equipment) [18]. We will also note the number of people moving past the equipment area without interacting with the equipment. Each site will be divided into targeted areas to be scanned. Systematic scans will be conducted over a 7-day period (Monday-Sunday) with a total of 11 scans per day as follows (22 and 55 scans for weekend and weekdays respectively): every 30 min of all park visitors in the study area during early morning (07:00–08:30), mid-day (11:30–13:00) and late afternoon (16:00–18:30) [19].

##### Intercept survey with visitors

Face-to-face intercept surveys (10–15 min) will be conducted at baseline (prior to site construction) and at 12 months follow up during the periodic observation (SOPARC). Trained, clearly identifiable research assistants will approach park users in the specified target areas, and will explain the study and all ethical considerations, and invite participation. Potential participants will be approached and provided with a verbal explanation about the study. Interested participants will then be asked to provide verbal agreement (consent) to participate. If there is more than one person in the park, the research staff will aim to recruit an equal number of men and women. The participants will be eligible to complete only one intercept survey during each test period.

Intercept surveys will provide more in depth information about park users’ characteristics. The survey will include a set of questions across various domains similar to previous research [19, 20] as well as validated questionnaires (detailed below). The set of questions will include socio-economic and demographic characteristics of park users (e.g., age group, gender, if born in Australia, marital status), if they are local residents or visitors, motivation to use the exercise park, how often they visit the park, social connectedness/engagement with other people at the park, their general physical activity level and their leisure/recreation activity at the park, and general health and well being. The following validated questionnaires will be used as part of the survey:

*Self-reported physical activity*—will be measured using three questions from the Active Australia Survey (Australian Institute Of Health And Welfare, 2003 [21]), which assessed walking, moderate, and vigorous activity, plus an indicator of total activity. The Active Australia questions are valid and reliable and are recommended for use in Australian population-based research and will provide measures of time and frequency in light, moderate and vigorous physical activities [22]. The Active Australia questions can also provide a measure as an estimate of energy expenditure in MET-minutes per week.

*General wellbeing* will be assessed using the five-item World Health Organization (WHO-5) Wellbeing questionnaire [23, 24]. The WHO-5 measures psychological wellbeing and depressive symptoms using 5 simple questions. The raw score will be calculated to obtain a percentage score, which ranges from 0 representing the worst imaginable well-being and 100 representing the best imaginable well-being.

At the 12 months follow up intercept surveys will also include additional questions of park visitors’ participation in community events and general park attendance, perceptions of any changes to the park, usage of the exercise park equipment, and suggestions for future improvements to the park. Although the intercept survey will be completed anonymously, at the follow up time point (12 months), participants will be asked if they have completed it previously. The intercept surveys will be conducted at the same 7-day period of the SOPARC systematic scans.

##### Online access monitor platform – the ‘ENJOY-ME-APP’

An innovative e-monitor tracker platform (online Progressive Web Application—(‘*ENJOY-ME-APP’*)) will be designed and developed (Curve Tomorrow, Australia) to monitor the usage and access of the Seniors Exercise Park by visitors at each site. The online platform will include an online application incorporating specific exercise instructions, videos, and safety tips. Quick Response (QR) codes will be placed on the instructional signage and on the exercise equipment itself at each site. Visitors will be able to scan the QR code with their mobile phone. Each QR code will have a unique identification code linked to a specific site. Each time a QR code is being used it will be registered on a secured cloud platform. Scanning the QR code from a mobile device will direct the user to a url address (similar to accessing website from a mobile device). No personal identification, or any sensitive information, and or mobile numbers will be recorded.

Visitors will be able to access the online platform at the site, scan the specific QR code and access the relevant video and associated information. The e-monitor tracker platform will monitor overall use collecting information of usage such as frequency, time and date. Design and testing of the e-monitor tracker platform will be conducted in the first 3–6 months of the project.

##### Review and audit of physical activity programs

Information about the type and number of physical activity programs for older people using the Seniors Exercise Park will be provided by the council (from the Positive Ageing team or equivalent) to the research team. There may be different modes of delivery and or programs that will be delivered by the participating partners and/or their respective local health/leisure providers. This information will be collected in the last stage of each site between 15 and 18 months, see Fig. 2.

### Seniors Exercise Park installation and site activation

#### The Seniors Exercise Park equipment installation

**Fig. 3:**
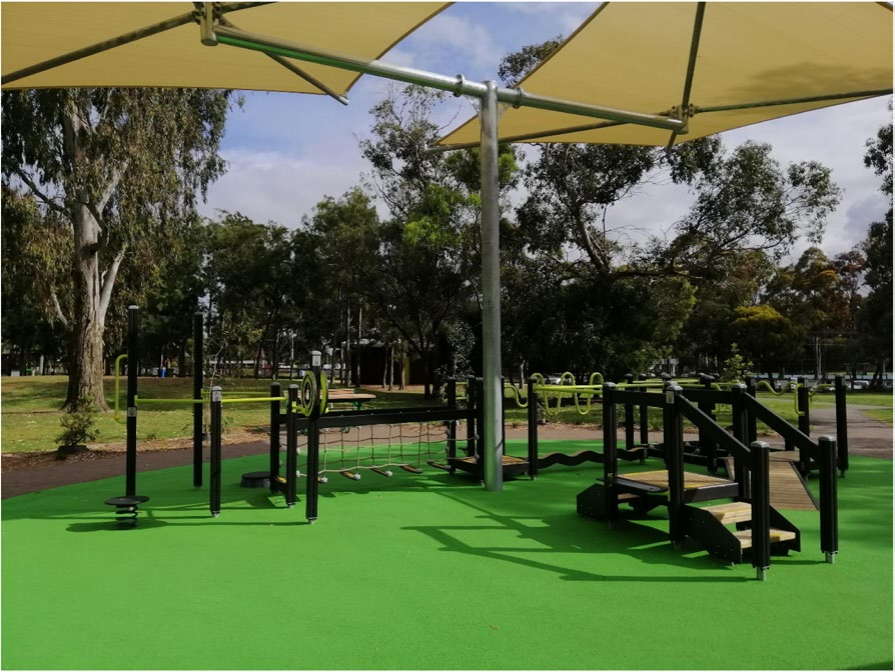
The Seniors Exercise Park at Thomas St Reserve, Hampton. The picture was taken by the lead investigator

The Seniors Exercise Park equipment is outdoor exercise equipment specifically designed for older people to improve strength, balance, joint movements and mobility and function (Fig. 3). It comprises multiple equipment stations that target specific function or movement (upper and lower limb) such as shoulder range of movement, static and dynamic balance, functional movement of walking up/down stairs, sit to stand. Councils will be provided with guidance around suitable flooring/surface (rubber surface or equivalent) and other safety measures for installation [9, 25]. The equipment is located outdoors and safe to use by various age groups (Fig. 3). Our previous multiple projects found the exercise park to be safe for older people (aged 60 years and over and with increased risk of falls) with no serious adverse events [11, 12].

#### Site activation

This project design and its implementation focus around sustainability. The ENJOY MAP for HEALTH incorporates four stages at each participating site with several strategies embedded to ‘activate a site’ to enable positive effects on physical activity behavior and sustainability: (1) promotion and marketing and (2) capacity building; both including development of resources. These will enable: ongoing promotion of physical activity opportunities, increased usage of the Seniors Exercise Park for community members, increased knowledge and community upskilling, and increased visitation to the park beyond the project completion.

1. Promotion and marketing

All four stages will be enhanced by ongoing communication and branding strategies and activities which will maximise visibility of the participating partners as well as increasing social/community connection. The branding strategy will include, but not be limited to, the design of promotional material to be distributed in online and off line channels such as digital (Facebook, Instagram, website) and physical promotion (banner, posters, flyers) platforms. Community events (official park launch, come-and-try sessions) will also be organised to engage and reach community members.

2) Capacity building—upskilling and training– knowledge transfer

- Training modules will include training for allied health professionals (Training workshops) and Senior Champions (Train the Trainer).

This will take place in stage two at each site. Relevant resources will be developed to support the workshops and training including on-line resources (video materials) and written educational materials (e.g., manuals).

Training workshops for allied health professionals: A half day training workshop will be delivered at each site for local allied health professionals that will cover safe Seniors Exercise Park use, exercise prescription and program design using the Seniors Exercise Park, incorporating risk management, theoretical and practical sessions.

Train the Trainer module: ENJOY champions: The train to train module aims to train and qualify seniors/community members (older people from the community) to facilitate utilisation of the Seniors Exercise Park for their members/peers, and more widely in the local community. Using this approach maximises community engagement and physical activity participation of the wider community of older people. The ENJOY champions will also be actively involved in project and council events. Train the trainer module will include twice weekly training sessions for 5 weeks as follows: 9 practical training sessions using the Seniors Exercise Park (1.5 hours duration incorporating interactive teaching with hands-on demonstrations) and final theoretical session for 3 hours incorporating risk management, physical activity and healthy lifestyle tips. Adherence will be monitored using participation log.

2 b) Community health care and leisure centre providers engagement

We will work closely with the Positive Ageing and Disability and Community Development teams at each local government council to identify and develop relationships with local health care/leisure providers. This will enable engagement throughout the various project stages including involvement in training, project meetings, and community events. The long term engagement aims for health care and leisure centre providers to actively participate in the development and delivery of a service/program utilising the Seniors Exercise Park for older people.

##### COVID19 risks mitigation

The COVID19 pandemic presents a very challenging time for community and local governments with various restrictions that may be posed by the Australian government.

Potential delays to site construction (e.g., due to shortage of staff, shipping delays) and/or data collection may occur. We will work closely with the partners to accommodate flexibility in our approach and the project’s timeline will be adjusted based on site upgrade completion. We also anticipate that usage and access to the parks may be impacted by lockdown periods, hence data collection periods (observation and intercept interviews) may need to be adjusted to avoid periods where access to outdoor equipment is prohibited.

Cleaning and hygiene practice—Hand sanitizers will also be available for both staff and participants to use at the beginning and completion of each exercise session, or during exercise sessions if required. The equipment will be regularly cleaned using detergent solution/disinfectant spray or wipes.

Outdoor training and physical distancing—Most training sessions will be provided outdoors which reduce the risk of transmission. Physical distancing will be maintained during data collection where possible (observations and face-to-face intercept surveys) and during training sessions. Exercise sessions will run in small groups based on State Government advise. Where possible research meetings and trainings will run online.

### Statistical methods

#### Sample size estimation for intercept surveys

To our knowledge, there are no natural experimental studies that have investigated the usage of exercise equipment following an installation and site activation of specialized outdoor exercise equipment for older people. Studies investigating park visitors before and after park refurbishment (including upgrade of play spaces for children) reported an increase of 33% in park visitors (all ages combined, with 17.3% age between 21–59, and 1.8% age 60 +), with an increase of 112% engagement in moderate physical activity by children and adults [26]. An observational study conducted in NSW reported only 3.8% (*n* = 112) of all visitors utilized outdoor exercise equipment for the purpose of exercise, among these approximately 5.3% (*n* = 6) were older people [18]. Another study reported only 15.6% of park users were older people (60–70 years), with people aged 60–70 being 60% less likely to report being regular park users than the younger age groups [27].

A previous study reported approximately 3 people/visitors every 1.5 h attending a community park [16]. With 3 periods of observations/ scans and total of 5.5 observation hours (early morning, mid-day, late afternoon) per day (estimated 12 people/visitors per day), we then estimate 84 visitors for the 7-day period. Based on a previous study, approximately 15.6–17.9% are expected to be older people [26, 27], hence a sample of 15 older people (17% of 84 visitors) will be expected to be recruited per site at the baseline observation period. With an estimated twofold increase in park visitors following site activation, a sample of 30 face to face intercept interviews will be targeted at the 12 month follow up observation period at each site. With potential 18–20% declining to participate (refusal), we will aim for a total of 38 intercept interviews (13 and 25 at baseline and follow up respectively, which accounts for potential refusals) per site with an overall sample of 228 interviewees across the six sites.

To determine the required sample size, it was assumed that the number of older people visiting the park per week would follow either a Poisson distribution, where the variance equals the mean (no overdispersion), or a negative binomial distribution, where the variance exceeds the mean (overdispersion) [28]. A baseline mean of approximately 15 per week was assumed, corresponding to a coefficient of variation (CV = standard deviation as percentage of mean) for the Poisson distribution of 25.8%. For the negative binomial, the CV was set at 38.7% (a 50% increase in relative variability). In addition, seasonal adjustments to the expected means were made, since it was likely the number of older people visiting the park will be impacted by weather [29]. Fake data simulation was used to determine the power [30]; 10,000 simulated data sets were generated using the Poisson and Negative Binomial distributions assuming a two-fold increase in the number of older people visiting the park. For each data set, the model detailed in the statistical analysis section below was fitted to the data, and the intervention effect was statistically significant at the 5% level for all of the data sets with the Poisson distribution and for over 80% of the data sets with the negative binomial distribution.

#### A targeted outcome for ENJOY MAP for HEALTH project

A study investigating the impact of major renovations to playfields (mainly used for soccer and baseball) of two public parks as well as physical activity programming, reported a significant increase in visitation and overall physical activity (4-9 fold increase) compared to the controls [31]. This suggests that intervention that includes change in the physical built environment complemented with other community engagement is likely to result in substantial increase in physical activity participation. With limited studies reporting older people’s usage and engagement in physical activity, we estimate a twofold increase in visits by older people from baseline to the 12 months follow up at each site. Hence, we hypothesise that the intervention (Seniors Exercise Park installation and site activation) will result in *at least* twofold increase (100%) in the number of visitors (older people) between baseline to the 12 months follow up.

#### Statistical analysis

Descriptive statistics will be used to report the overall park observation visitors, visitors’ characteristics, prevalence of usage of the Seniors Exercise Park, the type and level of physical activity of visitors (SOPARC). Descriptive statistics will also be used for the intercept surveys data to collate information about visitors’ perceptions about the park, motivation to use it, social connectedness/engagement with other at the park, their physical activity level and general wellbeing and their leisure/recreation activity at the park.

A generalised linear model [28] will be used to examine the effects of park refurbishment (equipment installation and site activation) on the total number of older people observed in the park, and the number of people walking and being physically active in the park. The statistical model will include terms for the intervention effect, the site effects, and seasonal effects; and overdispersion will be handled by using a quasipoisson family [32]. Statistical analyses will conducted using IMB SPSS Statistics version 26 (IBM Corporation, Armonk, NY, USA) and R [33].

## Discussion

The World Health Organisation Global Age-friendly Cities guide identifies outdoor space and building as one of the domains for cities and communities to deliver better outcomes for older people [34]. Age-friendly environments can foster healthy and active ageing by enabling accessible and age-appropriate safe environments. Designing age-friendly recreational outdoor spaces can promote health and health-related benefits at the population level [35–37] and promote physical activity of older people [27, 38].

The ENJOY MAP for HEALTH project is an active ageing project designed to fulfil the need for an age-friendly outdoor space for older people to support them to be physically active in the community. Interventions that involve the use of physical activity programs combined with a physical change to the built environment are likely to have a positive effect on physical activity [39]. The ENJOY MAP for HEALTH incorporates these aspects; it involves the upgrade of outdoor space with installation of specialised equipment and activities to maximise usage of the equipment by older people. Hence through our proposed four-stage process and partnership with local governments and community we aim to promote public health by combining built environment change and physical activity promotion intervention. Importantly, this project is very timely as it addresses the need for safe outdoor spaces for older people to be physically and socially active for their wellbeing. This is of particular importance in light of the anticipated recovery from the COVID19 pandemic as green space and outdoor spaces are well recognised for their importance for population wellbeing and health [40]. The project is expected to result in a significant change in the physical outdoor environment for the participating councils and communities whereby older people and other community members will be able to engage in safe physical and social activity programs, socialise more and hence improve the overall wellbeing of older people.

## Data Availability

Not Applicable.

## Abbreviations

ENJOY: Exercise interveNtion outdoor proJect in the cOmmunitY
ENJOY MAP for HEALTH: Exercise interveNtion outdoor proJect in the cOmmunitY - More Active People for HEALTHier communities
SOPARC: System for Observing Play and Recreation in Communities
WHO-5: The five-item World Health Organization wellbeing questionnaire
